# First mitochondrial genome of *Euprosopia* sp. (Diptera: Platystomatidae)

**DOI:** 10.1080/23802359.2020.1715274

**Published:** 2020-01-20

**Authors:** Peng Hou, Liang Wang, Xin Li, Ding Yang

**Affiliations:** a College of Life Science and Bioengineering, Shenyang University, Shenyang, China;; b College of Plant Protection, China Agricultural University, Beijing, China

**Keywords:** Complete mitochondrial genome, Platystomatidae, *Euprosopia*, Phylogenetics

## Abstract

Platystomatid flies, one of the largest families in Diptera. Here, we determined the complete mitogenome of *Euprosopia* sp., which is the first for the family Platystomatidae. The 16,266 base pair (bp) mitogenome comprises of 13 protein-coding genes (PCGs), 22 transfer RNA genes (tRNAs), 2 ribosomal RNA genes, and a control region. The gene order and the orientation are similar to those of other sequenced Acalyptratae species except ${\mathrm{tRNA}}^{\mathrm{Ser}(\mathrm{AGN})}$ gene were substituted by another ${\mathrm{tRNA}}^{\mathrm{Phe}}$ gene. All PCGs start with ATN codons, except *COI* and *ND1*, and end with TAA or incomplete stop codon TA + tRNA. The 22 tRNAs have a typical cloverleaf secondary structure. Phylogenetic analyses base on 13 Diptera species supported the monophyly of Superfamily Tephritoidea.

Platystomatid flies, one of the largest families of Acalyptratae flies, are almost distributed world-wide. It has the greatest number of species in the Old World tropics, particularly in New Guinea and tropical Africa, where many large, conspicuously marked species occur. There are around 119 known genera and nearly 1200 described species (McAlpine 2001). The biology of this family is rarely been studied (Ferrar 1987; Martínez-Sánchez et al. 2000). Adults of many species inhabit forests, while others live in sand dune and other vegetation types, some are attracted to fresh mammalian or other feces, some are recorded feeding at sap flows on injured trees and banana plants and sometimes at flowers, broken melons and other fruits, and the secretions of aphids.

Although three autapomorphies that prove monophyly of this family, none of them can be accepted without reservation (McAlpine 1989) and the monophyly of this family was not recovered in the molecular study by Han and Ro (2005). The previous phylogeny researches on high-level Tephritoidea have not been in agreement, and studies about relationships among genus and subfamily of this family are limited.

Genomic DNA was extracted from tissue samples using TIANamp Micro DNA Kit (Tiangen Biotech Co., Ltd). The library was sequenced on an Illumina HiSeq 2500. The bait sequence *COI* was amplified by standard PCR reactions, BLAST search was carried out with BioEdit 7.0.5.3. and the position of all tRNA genes was confirmed using tRNAscanSE 2.0 (Lowe and Chan 2016). Phylogenetic analysis was performed based on the nucleotide sequences of 13 PCGs. There are other 12 species were included in phylogenetic analysis. Using default parameters and 1000 bootstrap replicates, we constructed a phylogenetic tree based on maximum likelihood (ML) analysis by RAxML v0.6.0, which is available from the RAxML BlackBox (https://raxml-ng.vital-it.ch/#/).

Specimens of *Euprosopia* sp. were collected in Beibeng village of Tibet (29°14′4″N, 95°9′12″E) by Qicheng Yang, and identified by Liang Wang and Prof. Ding Yang. Specimens (CAU-HP-2019005) are deposited in the Entomological Museum of China Agricultural University (CAU). The complete mitochondrial genome of *Euprosopia* sp. (MK640609) was 16,226 bp in length, and consisted of 13 typical invertebrate PCGs, 22 transfer RNA genes, 2 rRNA genes (*12S* and *16S*), and a control region, the order of majority mitochondrial genes of *Euprosopia* sp. were similar with related reports before (Kang et al. 2016; Li et al. 2016; Wang, Ding, et al. 2016; Wang, Wang, et al. 2016; Wang, Liu, et al. 2016; Li et al. 2017; Zhou et al. 2017), except ${\mathrm{tRNA}}^{\mathrm{Ser}(\mathrm{AGN})}$ gene was substituted by another ${\mathrm{tRNA}}^{\mathrm{Phe}}$ gene.

The nucleotide composition of the mitogenome was biased toward A and T, with 73.0% of A + T content (A = 40.3%, T = 32.7%, C = 16.3%, G = 10.6%). It has 16 intergenic spacer lengths from 1 to 22 bp. There are 8 overleaping regions, with overlap lengths ranging from 1 to 8 bp. Among the protein-coding genes, 6 genes took the start codon of ATG and 5 genes used ATT as start codon, while *COI* gene and *ND1* gene got TCG and GTT, respectively. The termination codon of these protein-coding genes had three types (six genes were TAA, three genes were TAG, four genes use incomplete stop codon TA + tRNA). The longest gene in this mitochondrion was *ND5* (1720 bp), and the 22 tRNA genes ranged from 62 (${\mathrm{tRNA}}^{\mathrm{Cys}}$) to 72 (${\mathrm{tRNA}}^{\mathrm{Val}}$) in length. The *12S* rRNA (786 bp) and *16S* rRNA (1,326bp) were separated by ${\mathrm{tRNA}}^{\mathrm{Val}}$ gene. The putative control region (1394 bp) was also located between *12S* rRNA and ${\mathrm{tRNA}}^{\mathrm{Ile}}.$

ML analysis (Figure 1) supported the monophyly at the level of superfamilies, 4 Acalyptratae families each was a monophyletic group. Tephritoidea was supported as a monophyletic clade, but the bootstrap value was not high and family Tephritidae was not recovered as monophyletic lineage.

**Figure 1.**
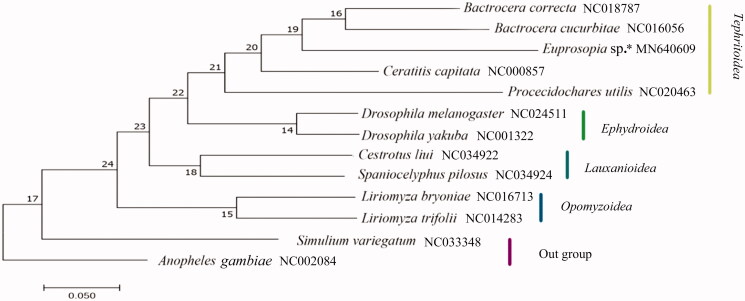
The phylogenetic tree of maximum likelihood analysis based on 13 PCGs. *Indicates this study.

The complete mitochondrial genome of *Euprosopia* sp. provides valuable relevant information to posterior genetic and evolutionary studies of *Euprosopia* genus and the Platystomatidae family.
